# Assessment of quality of obstetric care in Zimbabwe using the standard primipara

**DOI:** 10.1186/s12884-018-1863-5

**Published:** 2018-06-04

**Authors:** Bothwell Takaingofa Guzha, Thulani Lesley Magwali, Bismark Mateveke, Maxwell Chirehwa, George Nyandoro, Stephen Peter Munjanja

**Affiliations:** 1 Department of Obstetrics and Gynaecology, University of Zimbabwe, College of Health Sciences, P.O. Box A178, Avondale, Harare, Zimbabwe; 2 Department of Medicine, Division of Clinical Pharmacology, University of Cape Town, K45 Old Main Building, Groote Schuur Hospital, Observatory, Cape Town, 7925 South Africa; 3 Biomedical Research and Training Institute, 10 Seagrave Avenue, Avondale, Harare, Zimbabwe

**Keywords:** Standard primipara, Quality of care, Obstetric process indicators, Obstetric outcome indicators, the perinatal mortality rate

## Abstract

**Background:**

To improve maternity services in any country, there is need to monitor the quality of obstetric care. There is usually disparity of obstetric care and outcomes in most countries among women giving birth in different obstetric units. However, comparing the quality of obstetric care is difficult because of heterogeneous population characteristics and the difference in prevalence of complications. The concept of the standard primipara was introduced as a tool to control for these various confounding factors. This concept was used to compare the quality of obstetric care among districts in different geographical locations in Zimbabwe.

**Methods:**

This was a substudy of the Zimbabwe Maternal and Perinatal Mortality Study. In the main study, cluster sampling was done with the provinces as clusters and 11 districts were randomly selected with one from each of the nine provinces and two from the largest province. This database was used to identify the standard primipara defined as; a woman in her first pregnancy without any known complications who has spontaneous onset of labour at term. Obstetric process and outcome indicators of the standard primipara were then used to compare the quality of care between rural and urban, across rural and across urban districts of Zimbabwe.

**Results:**

A total of 45,240 births were recruited in the main study and 10,947 women met the definition of standard primipara. The maternal mortality ratio (MMR) and the perinatal mortality rate (PNMR) for the standard primiparae were 92/100000 live births and 15.4/1000 total births respectively. Compared to urban districts, the PNMR was higher in the rural districts (11/1000 total births vs 19/ 1000 total births, *p* < 0.001). In the urban to urban and rural to rural districts comparison, there were significant differences in most of the process indicators, but not in the PNMR.

**Conclusions:**

The study has shown that the standard primipara can be used as a tool to measure and compare the quality of obstetric care in districts in different geographical areas**.** There is need to explore further how the quality of obstetric care can be improved in rural districts of Zimbabwe.

## Background

The previous millennium development goal (MDG) number 5 targeted to reduce maternal deaths by 75% by the year 2015. This unrealistic target was not achieved because there is still a great need for unrestricted access to high-quality emergency obstetric care to reduce the high risk of dying in pregnancy which is still prevalent in the low-resource countries [1]. Thus reducing this great risk of dying during pregnancy in low-resource countries is the new target in the developed sustainable development goal (SDG) number 3, targeting to reduce the global maternal mortality ratio to less than 70 per 100,000 live births by 2030.

The lifetime risk of dying due to pregnancy-related complications in Sub-Saharan Africa is 1 in 39 compared to 1 in 4600 in the United Kingdom and 1 in 3800 in high-income countries in general [2].

To improve maternity services, there is need to monitor and improve the quality of care women receive in different obstetric units. One limitation though is that there is no universally agreed definition of quality of care (QoC). This explains the variation in obstetric outcomes like caesarean section, instrumental delivery and induction of labour rates among different health institutions within the same setting [3]. In most countries, there is a disparity of care and outcomes among different obstetric units [4]. For this to be corrected there is need to come up with a tool that can be used to compare the quality of care between the different institutions. The tool should be able to control for confounding factors like difference in patient characteristics and disease patterns.

In Zimbabwe, like in most countries, the difference in the quality of care among the different geographical areas is difficult to determine because of heterogeneous population characteristics and the difference in prevalence of complications in those areas. The concept of the standard primipara was introduced as a basis of inter-unit comparison in evaluating the quality of obstetric services to minimise the risks of bias [4]. Alfirevic et al. used this concept in comparing the impact of delivery suite guidelines on intrapartum care among different health institutions [5]. Some maternal and perinatal process and outcome indicators were then used as tools to compare the quality of obstetric care among the different geographical districts in Zimbabwe [6, 7].

The availability of comprehensive obstetric care in various settings depends on local conditions and the resources available. There is no available data looking at the quality of obstetric care among the different districts in the different Provinces of Zimbabwe. This study was done to identify the districts with poor quality of obstetric care. This data is important to improve maternity services in poorly performing districts to match those of areas that had better performance.

## Methods

Zimbabwe is divided into 10 administrative Provinces, which are divided into 59 Districts. Harare, the biggest Province has urban districts only unlike all the other Provinces which are comprised of urban and rural districts. The Zimbabwe Maternal and Perinatal Mortality Study (ZMPMS) was a population-based descriptive and cross-sectional study of deaths of women in pregnancy and perinatal deaths in Zimbabwe. The study was done to estimate the maternal mortality ratio (MMR) and the perinatal mortality rate (PNMR) in Zimbabwe [8]. Data were collected from the 1st of May 2007 to the 30th April 2008. Cluster sampling was done with the 10 provinces as clusters and 11 districts were randomly selected with one from each of the 9 provinces and 2 from Harare which is the biggest province in Zimbabwe. In these 11 districts, pregnancy outcomes were collected prospectively on all women delivering after 22 weeks gestation for 11 months [8]. Data were collected from all healthcare facilities and also from homes and villages. A data entry template was designed in Microsoft Access and used for data capture. Alfirevic et al. defined the standard primipara as a woman in her first pregnancy, with a singleton fetus in cephalic presentation, with spontaneous onset of labour between 37 + 0 weeks and 42 + 0 weeks, with no antenatal complications or previous hospital admission lasting more than 24 h [5]. This was the definition of the standard primipara used in this study. Data from all the districts in the main study were used to extract records for women who met the definition of the standard primipara; and subsequent data analyses were performed in Stata Version 9.0 (StataCorp LP, College Station, TX). The standard primiparae were then used to compare maternal and perinatal process and outcome indicators between rural and urban, across urban and across rural districts. Pearson chi-squared test was used to determine the association between the categorical variables. The quality of obstetric care was assessed using the following indicators:

a) Obstetric process indicators:Booking status (at least one antenatal visit), gestational age at booking, antenatal human immunodeficiency virus (HIV) screening rate, and initial place of onset of labour, utilisation of maternity waiting shelters in the rural districts, institutional delivery rate, intrapartum complication detection rate, and referral in labour rate, operative vaginal delivery rate, caesarean section rate and the postpartum referral rate.

b) Obstetric outcome indicators:PNMR.

## Results

In the main study, a total of 45,240 births were recruited from the 11 districts and 10,947 women met the definition of standard primipara.

As shown in Table 1 below, the median (Q1; Q3) age of the women was 20 (18; 22) years.

**Table 1:** Demographic characteristics of the standard primiparae, stratified by district

District/ Area	Sample size	Median age (years) (Q1; Q3)
Rural		
Bindura	837	19 (18;21)
Chivi	1394	20 (18;22)
Matobo	617	19 (17;20)
Mutoko	778	19 (18;21)
Tsholotsho	648	18 (17;20)
Zvimba	1005	20 (18;22)
Urban		
South Eastern district of Harare	444	23 (20;25)
Western district of Harare	1463	21 (19;23)
Kwekwe	1398	20 (18;23)
Nkulumane	1159	21.(19;23
Mutare	1204	20 (19;23)
Area		
Rural	6026	19 (18;21)
Urban	4921	21 (19;23)
Total	10,947	20 (18; 22)

As shown in Table 2 below, the vast majority of the standard primiparas booked their pregnancies (94.1%) and the median (Q1; Q3) gestation at booking was 24 (20; 28) weeks. Less than half of them (42.4%) were screened for HIV in the antenatal period. The institutional delivery rate was high at 87.8, and 8.8% of them were referred to higher levels of care for intrapartum complications. The caesarean section and vacuum delivery rates were low at 4.1 and 1.4% respectively. Compared to the rest of the women in the 11 districts, the standard primiparas had lower maternal mortality ratios (92/100000 live births vs 698/100000 live births, *p* < 0.001). The perinatal mortality rates were also lower in the standard primiparas (15.4/ 1000 total births vs 31.9/ 1000 total births, *p* < 0.001).

**Table 2:** Obstetric indicators for the standard primiparae

Process Indicators	Standard primipara *N* = 10,947
Booking status (%)	94.1
Mean gestation at booking (weeks)	23.6
Antenatal HIV screening (%)	42.4
Initial place of onset of labour-institutional (%)	19.4
Institutional deliveries (%)	87.8
Intra partum complications detected (%)	12.3
Referrals in labour (%)	8.8
Caesarean section rate (%)	4.1
Vacuum delivery rate (%)	1.4
Post-partum referrals (%)	5.5
Outcome Indicators	
PNMR (N/1000 births)	15.4

*Abbreviations*: *HIV* human immunodeficiency virus, *PNMR* perinatal mortality rate

As shown in the Table 3 below, in the urban to rural districts comparison, there were significant differences in the following process indicators: mean gestation at booking, antenatal HIV screening rate, initial place of onset of labour, institutional delivery rate, intrapartum complication detection rate, and referral in labour rate, caesarean section rate, vacuum delivery rate and post-partum referral rate. The urban districts had a significantly lower PNMR.

**Table 3:** Comparison between rural and urban districts

Process Indicators	Rural *N* = 6026	Urban *N* = 4921	*p-value
Booking status (%)	94.9	94.5	0.394
Mean gestation at booking (weeks)	22.4	25.3	< 0.001
Antenatal HIV screening (%)	37.8	60.1	< 0.001
Initial place of onset labour-institutional (%)	30.6	5.6	< 0.001
Institutional deliveries (%)	80.2	97.1	< 0.001
Intrapartum complications detected (%)	7.4	18.3	< 0.001
Referrals in labour (%)	3.1	15.8	< 0.001
Caesarean section rate (%)	2.2	6.5	< 0.001
Vacuum delivery rate (%)	0.5	2.5	< 0.001
Post-partum referrals (%)	0.4	1.2	< 0.001
Outcome Indicators			
PNMR (N/1000 births)	19	11	0.001

*Abbreviations*: *HIV* human immunodeficiency virus, *PNMR* perinatal mortality rate

*= Analysis of process and outcome indicators between districts

As shown in Table 4 below, across the urban districts, there were significant differences in the following process indicators: booking status, antenatal HIV screening rate, and initial place of onset of labour, institutional delivery rate, intrapartum complications detection rate, referral in labour rate, caesarean section rate, vacuum delivery rate and post-partum referral rate. There was no significant difference in the PNMR.

**Table 4:** Comparison across urban districts

Process Indicators	South Eastern district of Harare	Western district of Harare	Kwekwe	Mutare	Nkulumane	*p-value
Booking status (%)	95.7	90.7	96.8	96.5	95.4	< 0.001
Mean gestation at booking (weeks)	23.8	26.0	23.7	24.7	26.6	0.122
Antenatal HIV screening (%)	31.0	52.5	49.5	61.6	86.6	< 0.001
Initial place of onset of labour-institutional (%)	2.7	5.6	2.2	15.2	1.3	< 0.001
Institutional deliveries (%)	98.0	96.6	97.4	98.3	96.3	0.033
Intrapartum complications detected (%)	28.4	19.5	19.6	12.5	17.0	< 0.001
Referrals in labour (%)	9.5	16.7	14.1	23.4	12.1	< 0.001
Caesarean section rate (%)	8.4	5.0	9.8	5.7	5.9	< 0.001
Vacuum delivery rate (%)	15.9	2.0	0.5	0.1	1.5	< 0.001
Post-partum referrals (%)	1.6	1.6	0.7	0.1	2.3	0.001
Outcome Indicators						
PNMR (N/1000 births)	7	12	14	11	9.5	0.777

*Abbreviations*: *HIV* human immunodeficiency virus, *PNMR* perinatal mortality rate

*= Analysis of process and outcome indicators between districts

As shown in Table 5 below, across the rural districts, there were significant differences in the following process indicators: booking status, mean gestation at booking, antenatal HIV screening rate, initial place of onset of labour, institutional delivery rate, intrapartum complications detection rate, referral in labour rate and vacuum delivery rate. There was no significant difference in the PNMR.

**Table 5:** Comparison across rural districts

Outcome Indicators	Bindura	Chivi	Kwekwe	Matobo	Mutare	Mutoko	Zvimba	Tsholotsho	*p-value
Booking status (%)	95.3	97.3	97.6	98.0	96.1	96.8	98.1	84.0	< 0.001
Mean gestation at booking (weeks)	21.6	19.3	25.0	25.7	23.4	23.3	23.9	23.0	< 0.001
Antenatal HIV screening (%)	34.7	10.4	14.9	43.6	69.0	40.2	70.6	55.0	< 0.001
Initial place of onset of labour (%)	1.1	48.0	3.5	58.0	18.3	26.6	0.1	55.7	< 0.001
Institutional deliveries (%)	71.1	90.7	67.1	85.6	87.8	75.4	81.1	77.7	< 0.001
Intra partum complications detected (%)	4.1	4.4	6.9	10.3	2.7	16.2	3.9	9.3	< 0.001
Referrals in labour (%)	4.6	4.4	3.3	1.3	3.1	0.8	1.1	4.4	< 0.001
Caesarean section rate (%)	2.3	1.9	2.3	1.8	1.0	2.4	3.0	2.1	0.645
Vacuum delivery rate (%)	0.5	0.4	0.0	2.3	0.0	0.1	0.0	0.5	< 0.001
Post-partum referrals (%)	0.4	0.2	0.0	0.9	0.0	0.4	0.3	1.0	0.066
Outcome Indicators									
PNMR (N/1000 births)	24	22	18	20	31	14	22	10	0.234

*Abbreviations*: *HIV* human immunodeficiency virus, *PNMR* perinatal mortality rate and

*= Analysis of process and outcome indicators between districts

As shown in Table 6 below, the standard primiparas had significantly better obstetric and process outcome indicators than the general obstetric population.

**Table 6:** Comparison between the standard primipara and the general obstetric population

Process Indicators	Standard primipara *N* = 10,947	Non-Standard primipara *N* = 34,293	*p-value
Booking status (%)	94.1	76.7	< 0.001
Mean gestation at booking (weeks)	23.6	24.5	< 0.001
Antenatal HIV screening (%)	42.4	31.2	< 0.001
Initial place of onset of labour-institutional (%)	19.4	10.9	< 0.001
Institutional deliveries (%)	87.8	62.4	< 0.001
Intra partum complications detected (%)	12.3	11.7	0.091
Referrals in labour (%)	8.8	5.1	< 0.001
Caesarean section rate (%)	4.1	3.8	0.157
Vacuum delivery rate (%)	1.4	0.7	< 0.001
Post-partum referrals (%)	5.5	1.0	< 0.001
Outcome Indicators			
PNMR (N/1000 births)	15.4	31.9	< 0.001

*Abbreviations*: *HIV* human immunodeficiency virus, *PNMR* perinatal mortality rate

*= Analysis of process and outcome indicators between districts

## Discussion

Compared to the total ZMPMS population, the standard primiparas had a lower maternal mortality ratio and perinatal mortality rate (see Table 6). This confirms that this was a low-risk group and the differences in outcomes were probably due to variation in quality of service provision than patient-related factors.

Across urban and across rural districts (see Tables 4 and 5), there were significant differences in most of the obstetric process indicators. Some of the differences across the rural or urban districts were inexplicable considering that they get similar resources from the central Government. There is need to investigate why this is happening and offer remedial action to enable standardised maternity care in Zimbabwe. Surprisingly these differences did not have an impact on the PNMR. The study was not powered to detect a difference in the MMR among the individual districts.

In the rural to urban districts comparison (see Table 3), the only obstetric process indicator that did not show a significant difference was the antenatal booking status. The provision of free antenatal care in the Government rural clinics could explain why booking rates are high in both the rural and urban districts of Zimbabwe [9]. Despite these high booking rates, antenatal HIV screening rates remain low in both urban and rural districts (60.1% vs 37.8%, *p* < 0.001). This probably explains why a quarter of maternal deaths in Zimbabwe are still due to HIV/AIDS [8]. Zimbabwe has adopted the World Health Organisation (WHO) HIV guidelines which recommended that all HIV-infected pregnant women be put on anti-retroviral treatment regardless of their CD4+ or viral load count [10]. Therefore, there is need to put mechanisms in place to make sure that all pregnant women are screened for HIV and those infected are put on treatment to reduce the MMR and parents to child transmission rates of HIV.

More standard primiparae in the urban districts had access to skilled birth attendants (SBA) (97.1% vs 80.2%, *p* < 0.001) (see Table 3). The low rate of delivery by skilled birth attendants in the rural districts fell below the target of 90% set by the WHO [11]. Due to unavailability of transport, almost 20% of women ended up delivering outside institutions.

In the urban districts, the number of standard primiparas delivering in comprehensive emergency obstetric units and caesarean section rates meet the minimum targets set by the WHO of 15 and 5%, respectively [1, 12]. In the rural districts, the caesarean section rate of 2.2% is way below the WHO recommendation. Coupled with this, the operative vaginal delivery rate of 0.5% is way below the 2.5% in the urban districts. The low caesarean and operative delivery rates in the rural districts could be as a result of lack of personnel who are trained to do the procedures and a reluctance to attempt vacuum deliveries in remote areas, and this could explain the higher PNMR. Although the Central Government through the Ministry of Health and Child Care (MOHCC) has started to enforce the policy of deploying recently qualified doctors to the rural districts, this might not have an impact in the long term unless maternity waiting shelters are fully utilised to improve the institutional delivery rates in these areas.

## Conclusions

The study has shown that the standard primipara is a useful tool to measure the quality of obstetric care in different districts in Zimbabwe**.** Therefore every district should measure process and outcome indicators of the standard primipara. The MOHCC can use this tool to monitor improvement in obstetric care and to find out the specific reasons for the discrepancy in the different obstetric process indicators and how this variation in service provision can be corrected at local and national level.

To the best of our knowledge, no study has been done in Africa utilising this tool to measure the quality of obstetric care between institutions in one country. A pilot study can also be done to assess its feasibility in comparing the quality of obstetric care among different countries in Africa.

## Abbreviations

AIDS: Acquired Immunodeficiency Syndrome
HIV: Human Immunodeficiency Virus
MDG: Millennium Development Goal
MMR: Maternal Mortality Ratio
MOHCC: Ministry of Health and Child Care
PNMR: Perinatal Mortality Rate
QoC: Quality of Care
SBA: Skilled Birth Attendant
SDG: Sustainable Development Goal
WHO: World Health Organization
ZMPMS: Zimbabwe Maternal and Perinatal Mortality Study
